# **Exogenous γ-aminobutyric acid improves the photosynthesis efficiency, soluble sugar contents, and mineral nutrients in pomegranate plants exposed to drought, salinity, and drought-salinity stresses**

**DOI:** 10.1186/s12870-023-04568-2

**Published:** 2023-11-06

**Authors:** Saeedeh Zarbakhsh, Ali Reza Shahsavar

**Affiliations:** Department of Horticultural Science, College of Agriculture, Shiraz University, Shiraz, Iran

**Keywords:** Compatible osmolytes, Drought-salinity stress, GABA, Growth indices, Mineral nutrients, Photosynthetic capacity, Pomegranate

## Abstract

**Background:**

γ-aminobutyric acid (GABA), as a regulator of many aspects of plant growth, has a pivotal role in improving plant stress resistance. However, few studies have focused on the use of GABA in increasing plants’ resistance to interactional stresses, such as drought-salinity. Therefore, the focus of this study was to examine the effect of foliar application of GABA (0, 10, 20, and 40 mM) on growth indices and physio-biochemical parameters in plants of two pomegranate cultivars, ‘Rabab’ and ‘Atabaki’ exposed to drought, salinity, and drought-salinity.

**Results:**

Under stress conditions, the photosynthetic capacity of two pomegranate cultivars, including transpiration rate, net photosynthetic rate, intercellular carbon dioxide concentration, stomatal conductance of water vapour, and mesophyll conductance, was significantly reduced. This resulted in a decrease in root morphological traits such as fresh and dry weight, diameter, and volume, as well as the fresh and dry weight of the aerial part of the plants. However, the application of GABA reversed the negative effects caused by stress treatments on growth parameters and maintained the photosynthetic capacity. GABA application has induced the accumulation of compatible osmolytes, including total soluble carbohydrate, starch, glucose, fructose, and sucrose, in charge of providing energy for cellular defense response against abiotic stresses. Analysis of mineral nutrients has shown that GABA application increases the absorption of potassium, potassium/sodium, magnesium, phosphorus, manganese, zinc, and iron. As concentration increased up to 40 mM, GABA prevented the uptake of toxic ions, sodium and chloride.

**Conclusions:**

These findings highlight the potential of GABA as a biostimulant strategy to enhance plant stress tolerance.

## Background

Pomegranate (*Punica granatum* L.) has been appreciated for centuries as one of the oldest and most commercially edible horticultural fruit trees throughout the world. This remarkable fruit has high medicinal benefits and nutritional value because of its high antioxidant activity and bioactive phenolic compounds [1]. Pomegranate has been widely cultivated across many countries, particularly in the arid and semiarid regions of Iran [2, 3]. However, in these regions, pomegranate plants are often faced with the significant challenge of drought stress and the evaporation caused by drought stress has led to an increase in soil salinization. Consequently, the combined impact of drought and salinity stress can significantly affect both the yield and quality of pomegranate fruits.

When plants are subjected to water deficit conditions, the accumulation of ions, predominantly Na, in the soil’s upper layers causes osmotic stress and ion toxicity, nutritional shortage, and/or a combination of these factors, which are the most important negative effects of these stressful conditions on plant growth and development [3–5]. Globally, it has been reported that drought stress and soil salinization negatively affect 40% and 11% of the world’s total irrigated land, respectively [6, 7]. Furthermore, it is estimated that about half of the arable land will be salinized by 2050 [5]. The impact of the abiotic stresses on plants natural growth depends upon its severity, duration, plant species, and morphological phases of plants. However, water deficits and salinity from soil or irrigation disrupt the water availability of plants, causing the plant cells to become dehydrated. Under the mentioned unfavorable conditions, stomatal closure prevents water loss as well as restricts leaf CO_2_ diffusion, which eventually limits photosynthesis and inhibits carbon assimilation metabolism during dark reactions [8, 9]. In addition, the excess of light absorbed from the sun not used in a plant’s photosynthesis can lead to the production of reactive oxygen species (ROS), and consequently, this photochemical impairment leads to oxidative stress [4, 9]. Plants use specific mechanisms to rescue themselves from the negative consequences of drought and salinity. One of the mechanisms is to accumulate compatible organic solutes such as carbohydrates, amino acids, and nitrogen that play an important role in minimizing the harmful effects of ROS and protecting membranes and proteins by reducing cell osmotic potential, increasing accumulating capacity, and maintenance of cell water. Moreover, compatible solutes enable the plant to maintain cell turgor, increasing secondary metabolites and preventing the absorption of toxic ions sodium (Na) and chloride (Cl) [10]. As the most common osmoprotectant, soluble sugars participate in the diverse physiological activities of plants and play a major role in abiotic stress response, osmoregulation, signaling, and intermediating for the synthesis of organic molecules and carbon-storing compounds [11]. Furthermore, soluble sugars not only act as a carbon skeleton for the synthesis of defense compounds, including secondary metabolites including flavonoids, stilbenes, and lignin but also act as energy sources and precursors used in the respiratory processes that provide energy for cellular defense responses against abiotic and biotic stresses [12].

However, research focused on the combined effects of abiotic stresses on plants has indicated that it is not possible to predict the combined effects of these stresses based on plant responses to individual stresses. It is thus important to understand the different controlling mechanisms in the field of tolerance to combined drought and salinity stress, especially on a large scale; however, there is still a knowledge gap about stress combinations. Since global climate change is increasing at an alarming rate, the time frame for developing resistance in plants to stress combinations has been shortened. It is therefore vital to increase the drought and salinity resistance of pomegranate plants through economic and effective strategies. Extensive mitigation and adaptation approaches have been applied to the development of plant resiliency against various biotic and abiotic stresses through economic and effective strategies, including the application of exogenous plant growth regulators (PGRs) such as γ-aminobutyric acid (GABA). GABA, as a natural non-protein amino acid has a metabolite role in plants. Moreover, GABA is signaling molecule and widely distributed in plants and can be involved in numerous processes, including the regulation of cytosolic pH, redox status, carbon (C) and nitrogen (N) metabolism, C/N fluxes, and acting as an osmoregulator [13]. Multiple previous investigations have revealed exogenous GABA function in regulating many aspects of plant growth and development [14]. Studies also have demonstrated that GABA has a pivotal role in ameliorating the negative effects caused by various environmental stresses on morphological and physiological traits of plants, including poplar (*Populus tomentosa* Carr.) and strawberry (*Fragaria*×*ananassa* Duch.) exposed to salt stress [15, 16], creeping bentgrass (*Agrostis stolonifera*) and mungbean (*Vigna radiata* L.) exposed to heat shock [17, 18], tomato (*Solanum lycopersicum* L.) exposed to low temperature [19], black cumin (*Nigella sativa* L.) exposed to deficit water [20], and maize (*Zea mays*) and *Prunus* rootstocks exposed to waterlogging stress [13, 21]. Moreover, research has demonstrated that GABA can produce compatible osmolytes (e.g., sugar, proline, trehalose, and polyamines) in response to drought or salinity stress in plants [20, 22–25]. Nevertheless, the effect of GABA on the regulation of mineral nutrition content in plants under stress conditions has received limited attention.

In our previous investigation, we found that exogenous GABA treatment, even at a low concentration of 10 mM, has drastically protected plants against oxidative stress by improving the some growth indices of pomegranate plants during periods of drought and salinity stress [26]. However, the effect of GABA on alleviating the oxidative injuries caused by soil drought and salinity stresses on pomegranate plants is associated with other comprehensive responses based on physiological and biochemical changes that have not yet been fully elucidated. Consequently, through the current study for the first time, we aim to investigate deeper into the impact of the exogenous application of GABA on plant responses at both the morphological (root morphology and plant growth) and physio-biochemical levels, specifically leaf gas exchange parameters, nutrient elements, and sugar contents of two popular pomegranate cultivars under drought and salinity stress conditions. Ultimately, we hope to broaden our perception of the adaptation and tolerance of pomegranate plants through the application of GABA exogenous treatment on different direct and indirect mechanisms under both individual and co-occurring drought and salinity stress treatments.

## Materials and methods

### Plant material, and growth conditions

Two pomegranate cultivars (*Punica granatum*) of two-year-old ‘Rabab’ and ‘Atabaki’ were considered as samples and transplanted into 10 kg plastic pots (40 cm × 24 cm) containing soil and leaf litter (3:2 w/w). Pomegranate plants were cultivated in the greenhouse of the Agriculture College at Shiraz University, Iran. Both cultivars had been grown for 4 months at 28 ± 1 °C temperature, relative humidity (RH) = 65 ± 5%, and 16/8 h light/dark photoperiod until exposed to stress treatments. Also, all plants were fertilized once a week using ½ Hoagland’s nutrient solution (pH 7.0) before the experiment.

### Experimental design and treatments

Pomegranate plants, during their vegetative growth stage, with the same height and growth rates when they have young and fully expanded leaves, were selected for the experiment. Afterward, plants were separated into four groups: (i) control; (ii) salinity stress (60 mM NaCl); (iii) drought stress (60% field capacity, FC); and (iv) salinity-drought stress (60 mM NaCl plus 60% FC). To control the drought stress treatment, the pot weighing method was employed. Dry soil weight was determined by placing 4 kg of soil in an oven at 103 °C for 48 h. The oven-dried soil was then used to fill the pots. The pots were thoroughly watered to saturate the soil, and the percentage of FC was calculated using the following equation:

1 $${\text{FC }}(\% ){\text{ }} = {\text{ }}\left( {\,\frac{{{\text{saturated}}\,{\text{soil}}\,{\text{weight}} - {\text{dry}}\,{\text{soil}}\,{\text{weight}}}}{{{\text{dry}}\,{\text{soil}}\,{\text{weight}}}}} \right) \times 100$$

The water content corresponding to FC was calculated by subtracting the weight of the dry soil and the pot. Accordingly, the level of drought stress (60% FC) was determined [27]. At the same time as exposure to stress treatments, GABA (Sigma Aldrich, St. Louis, MO, USA) in various concentrations (0, 10, 20, and 40 mM) was administered through leaf spraying. The GABA application was carried out using a manual pump, with a total volume of 50 ml of GABA solution being sprayed onto the leaves three times at 15-day intervals. GABA was dissolved in distilled water, and the choice of GABA concentrations was based on previous studies conducted on fruit plants [16, 21, 28, 29]. The stress experiment was performed for 45 days (between June 26 and August 11, 2021). For physiological analysis, fresh and fully developed leaves were collected, immediately frozen in liquid nitrogen, and stored at − 80 °C.

### Plant growth measurements

On the 45th day of stress treatments, whole plants were carefully removed from the ground and separated them into the aerial parts (i.e., stems and leaves) and roots, and washed repeatedly with deionized water. Growth and morphological traits, including root volume (cm^3^), root diameter (mm), and fresh and dry weights (FW and DW; g) of the plant aerial part and the root system, were measured. For measurement of dry weight, the plant aerial parts and roots were dried separately in an air-forced oven at 75 °C for 72 h.

### Leaf gas exchange measurements

The transpiration rate (Tr; mmol H_2_O m^− 2^ s^− 1^), net photosynthetic rate (Pn; µmol m^− 2^ s^− 1^), intercellular carbon dioxide concentration (Ci; µmol CO_2_ mol^− 1^), and stomatal conductance of water vapour (gs; mol m^− 2^ s^− 1^) values were measured using a portable photosynthesis system (LCi, ADC Bioscientific Ltd., Hoddesdon, Herts, England, UK) on a sunny day during 9:00–11:00 am, as described by Haworth et al. [30]. The environmental light intensity during the tests was 1200 µmol (photon) m^− 2^ s^− 1^, the leaf’s ambient temperature was 25 °C, the CO_2_ concentration was 400 µmol mol^− 1^, and the relative humidity ranged from 60 to 65%. Measurements were taken using the completely expanded leaves located in the upper third of each plant’s main stem. Stomatal limitation (Ls; %) was calculated as Ls = 1-Ci/Ca (Ca represents the ambient CO_2_ concentration) [31], and mesophyll conductance (Gm; mol CO_2_ m^− 2^ s^− 1^) was calculated as Gm = Pn/Ci [32].

### Sugar contents measurements

Total soluble carbohydrate (TSC) content was evaluated using the phenol-sulfuric acid method [33]. Accordingly, after drying the pomegranate leaves at room temperature, 0.1 g of dried and powdered leaf samples was extracted three times with 10 mL of 80% ethanol, followed by centrifugation for 10 min at 5000 rpm. Then, 1 mL of a 5% phenol solution was added to 1 mL of the supernatant, and immediately, 5 mL of concentrated sulfuric acid was added to the mixture and vigorously shaken for 30 s by a vortex machine to dissolve well. After cooling the contents of the test tube in an ice bath, the resulting color intensity was measured at a wavelength of 490 nm using an Epoch Microplate Spectrophotometer (BioTek Instruments, Inc., USA), and the results were expressed as mg g^− 1^ DW.

The content of three abundant soluble sugars (glucose, fructose, and sucrose) contents was extracted by the method of Kagan et al. [34]. The air-dried samples (0.1 g) were extracted in 50 mL of deionized water for 1 h at 40 °C, followed by centrifugation at 10,000 rpm for 10 min, and the supernatant filtered through 0.45 μm syringe filters. Subsequently, all extracted samples were analyzed by high-performance liquid chromatography (HPLC; Knauer, Germany) with a Eurokat Pb column. HPLC-grade distilled water with a flow rate of 1.0 mL min^− 1^ was used as the mobile phase. The column temperature was maintained at 65 °C. Finally, soluble sugar concentrations were identified and quantified by the refraction index (RI) detector, and the retention times of peaks were used to determine sugar contents based on their external analytical standards. The soluble sugars concentration was calculated by drawing calibration curves and expressed as mg g^− 1^ DW.

### Starch content determination

Upon completion of the TSC extraction process, the residual tissue obtained from the TSC was retained for the extraction of starch. To analyze starch content, 2 mL of distilled water was added to the tube containing the leaf material. The samples were vigorously shaken for 30 s using a vortex machine to dissolve well, followed by incubation at 100 °C for 15 min. Subsequently, the samples were allowed to cool to room temperature. Then, 9.2 M HClO_4_ (2 mL) was used to hydrolyze the leaf starch for a duration of 15 min. Following this, 4 mL of distilled water was added to the samples, which were then centrifuged at 5000 rpm for 10 min. The residue underwent an additional extraction step using 4.6 M HClO_4_ (2 mL). The supernatants were collected, combined, and diluted with distilled water to achieve a final volume of 25 mL was attained. Starch concentration was determined by the anthrone reagent at a wavelength of 630 nm. The concentration of starch was calculated in mg g^− 1^ DW [35].

### Determination of macro and micronutrients content

Mineral analyses were performed on dried leaf samples. Sodium (Na) and potassium (K) content were determined by the Flame photometer (JENWAY-P7P7 UK) [36]; phosphorus (P) content was estimated by colorimetric method [37]; and concentrations of magnesium (Mg), manganese (Mn), zinc (Zn), and iron (Fe) were determined by atomic absorption spectrophotometry (iCE 3500, Thermo Scientific, USA). The concentration of chloride (Cl) was determined according to the method of Mastrogiannidou et al. [38].

2 $${\text{Cl = }}\frac{{{\text{(mL\,AgNO}_{\text{3}}} - {\text{mL\,blank)}} \times {\text{N\,AgN}}{{\text{O}}_{\text{3}}} \times {\text{35}}{\text{.5}} \times {\text{100 }}}}{{{\text{g\,sample}} \times {\text{100}}}}$$

### Statistical analysis

This experiment was carried out in a completely randomized design (CRD) with a factorial arrangement. For each treatment, four independent replicates were applied. Data analysis was performed using SAS software (v9.4; SAS Institute, Cary, NC), and statistically significant differences among the treatments were determined using Fischer’s least significance difference (LSD) at the 5% probability level (*p* ≤ 0.05). Additionally, the experimental data were analyzed using principal component analysis (PCA-biplot) based on a correlation matrix using Minitab v16 statistical software. Pearson correlation was calculated to distinguish the correlation between dependent variables using the http://heatmapper.ca online program package.

## Results

### Morphological characteristics in response to drought and salinity stress alone and with GABA treatment

**Fig. 1:**
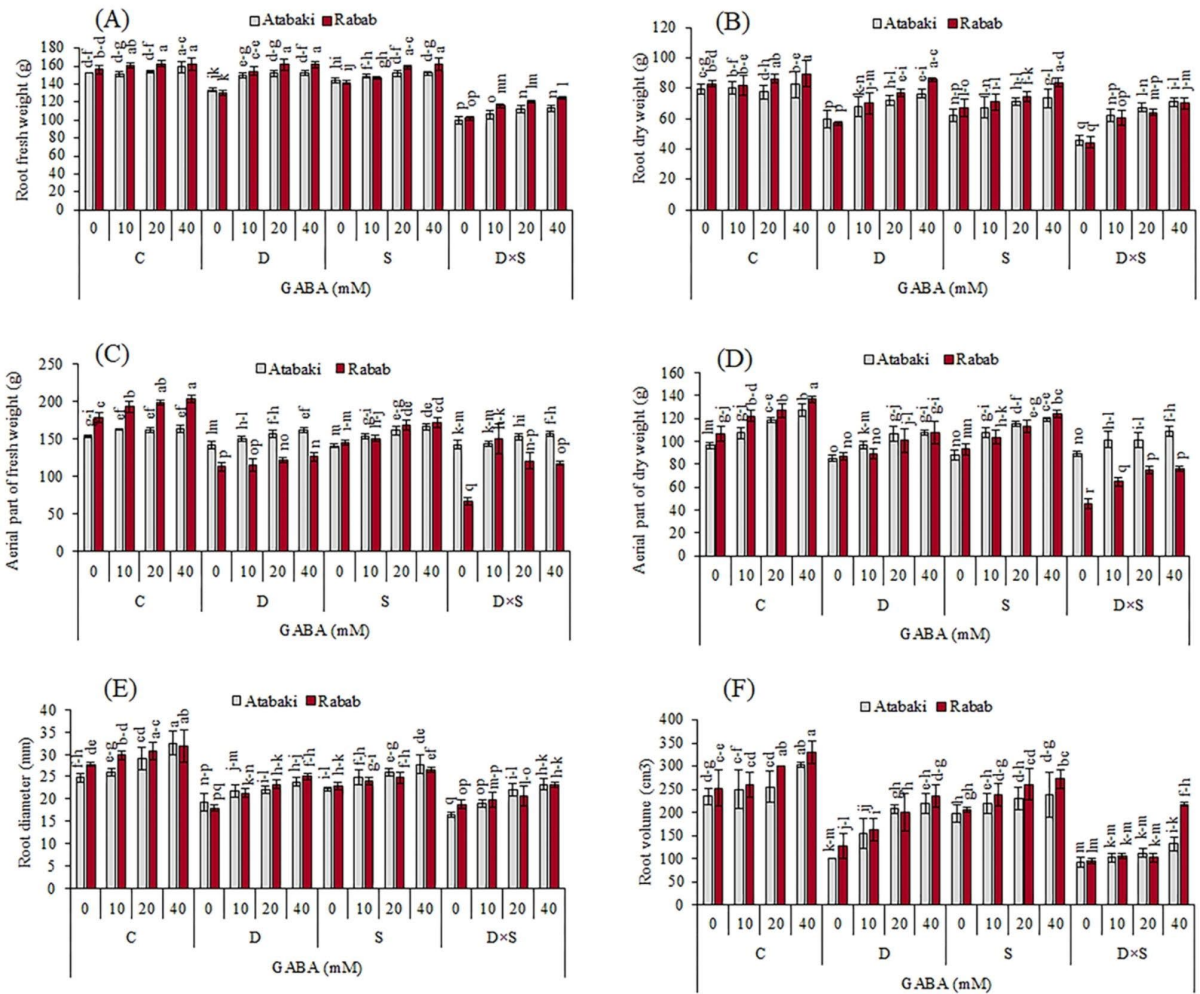
Effect of exogenous GABA on (A) root fresh weight, (B) root dry weight, (C) plant aerial part fresh weight, and (D) plant aerial part dry weight in pomegranate plants subjected to drought and salinity stresses. Control (C), drought (D), salinity (S), and drought-salinity (D×S). Vertical columns indicate mean ± standard deviation (SD)

**Fig. 2:**
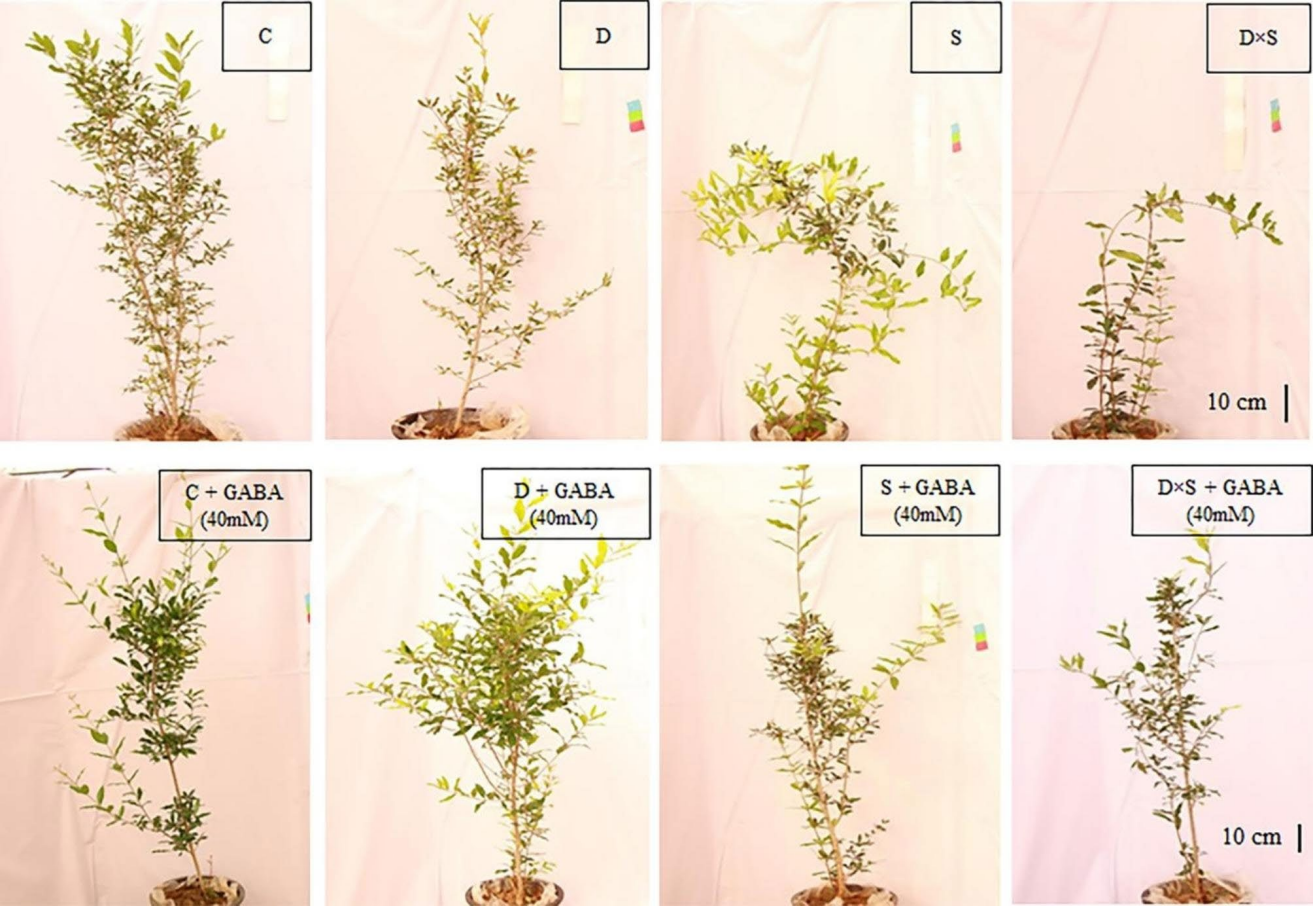
Monitoring of the morphological status of the pomegranate cultivar ‘Rabab’ under drought and salinity stress, where the upper part of the image indicates the pomegranate plants untreated and the lower part of the image indicates the pomegranate plants treated with a concentration of 40 mM GABA. Control (C), drought (D), salinity (S), and drought-salinity (D×S)

**Fig. 3:**
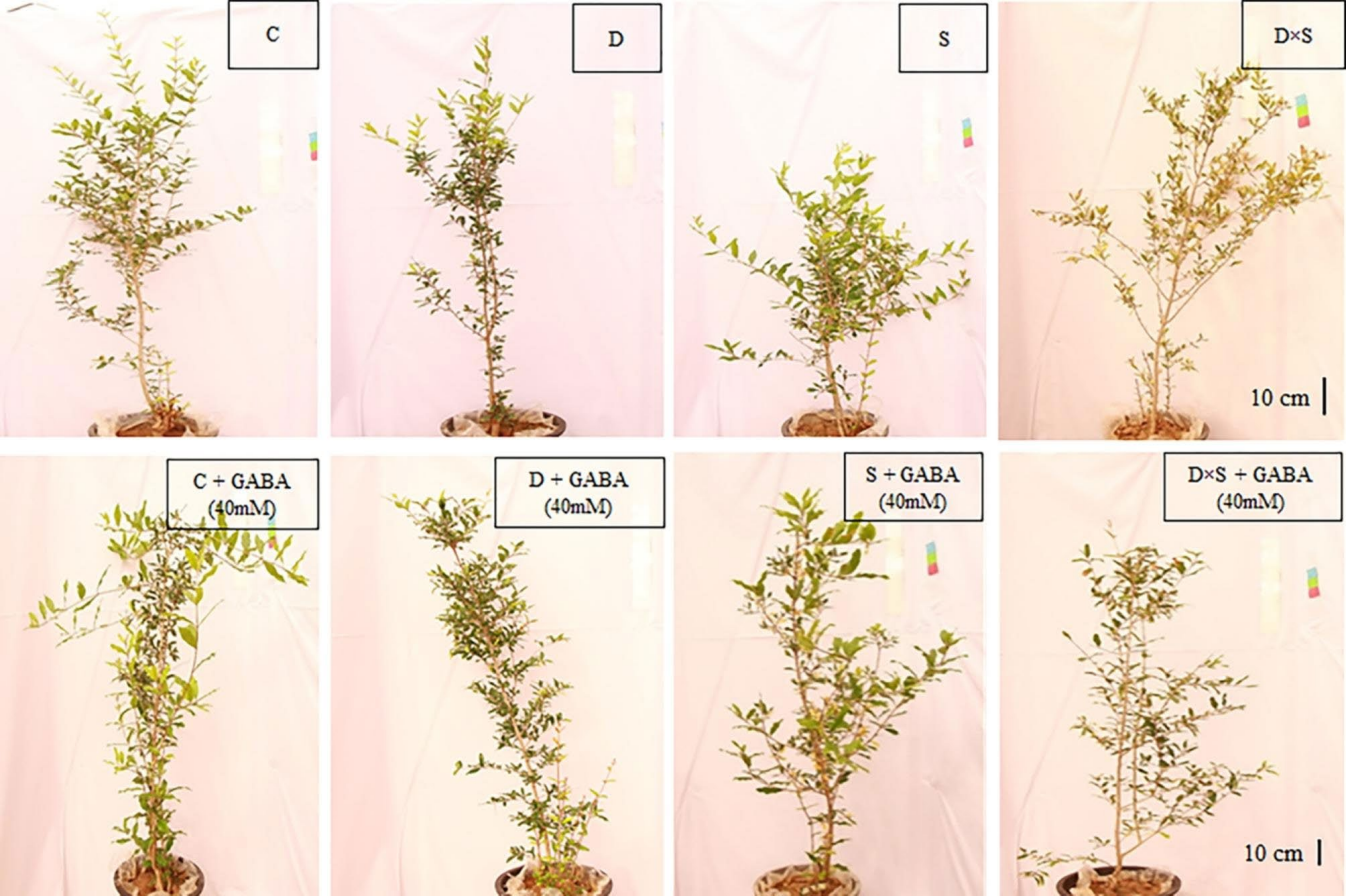
Monitoring of the morphological status of the pomegranate cultivar ‘Atabaki’ under drought and salinity stress, where the upper part of the image indicates the pomegranate plants untreated and the lower part of the image indicates the pomegranate plants treated with a concentration of 40 mM GABA. Control (C), drought (D), salinity (S), and drought-salinity (D×S)

As shown in Figs. 1 and 2, and 3, the morphological characteristics of the two cultivars were significantly affected by GABA application, drought, and salinity stress when compared with control plants. In root volume, root diameter, root and plant aerial part fresh weight, and dry weight, Fig. 1 also shows that these decreases were greater in plants treated with a drought and salinity combination than in plants exposed to only one stress. In this regard, compared with the stress and control treatments, the minimum value of the fresh and dry weight of the root was detected in plants exposed to the combination of drought and salinity stress, which showed 102 g and 44.25 g, respectively, in the ‘Rabab’ cultivar and 99.75 g and 45.75 g, in the ‘Atabaki’ cultivar (p ≤ 0.05). Similar changes were obtained under drought and salinity combinations in the fresh and dry weight of the aerial part of the ‘Rabab’ cultivar by 67 g and 45.5 g, respectively, and in the ‘Atabaki’ cultivar by 142 g and 88.75 g, respectively. Remarkably, the exogenous application of GABA treatment significantly increased the morphological characteristics of pomegranate plants under stress treatments, though the aerial part of the plants and root traits still showed weakness when compared with the control plants. In this regard, the highest root fresh and dry weight of plants was observed with 40 mM GABA foliar application under unstressed, drought, and salinity stresses in the ‘Rabab’ cultivar, and the highest aerial fresh and dry weight of plants was observed with 40 mM GABA foliar application under unstressed treatment in the ‘Rabab’ cultivar by 203 g and 136.5 g, respectively (Fig. 1). Similarly, the interaction between GABA and stress treatments had a significant effect on root traits; however, the maximum increase in root diameter and root volume was exhibited in the ‘Rabab’ cultivar by 31.75 mm and 330 cm^3^, respectively, and in the ‘Atabaki’ cultivar by 32.5 mm and 302.5 cm^3^, respectively.

### Effects of exogenous GABA and drought-salinity stress on photosynthesis and gas exchange parameters

**Fig. 4:**
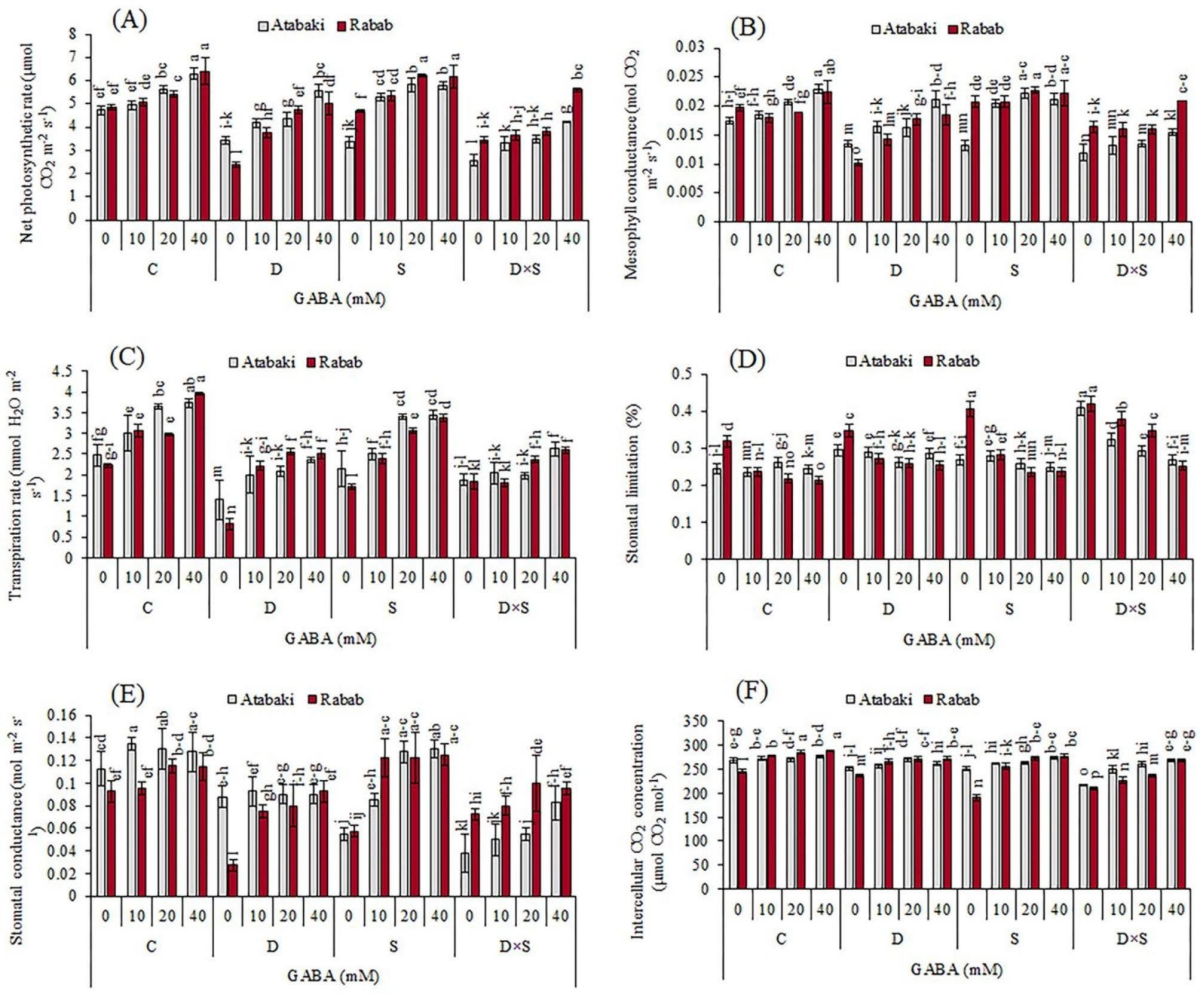
Effect of exogenous GABA on (A) net photosynthetic rate, (B) mesophyll conductance, (C) transpiration rate, (D) stomatal limitation, (E) stomatal conductance, and (F) intercellular CO_2_ concentration in pomegranate plants subjected to drought and salinity stresses. Control (C), drought (D), salinity (S), and drought-salinity (D×S). Vertical columns indicate Mean ± standard deviation (SD)

Results showed that both stress treatments (individual and co-occurring) significantly (p ≤ 0.05) decreased Pn, Tr, Gm, gs, and Ci of the two cultivars when compared with control plants (Fig. 4). The Ls increases significantly (p ≤ 0.05) under stress conditions; however, it is more outstanding among plants subjected to drought and salinity stress combined. Noticeably, foliar application of GABA in pomegranate plants exposed to stress significantly restored the effects of stress on these parameters and also improved the performance of these leaf gas exchange parameters as compared to the stress treatment without GABA application. In addition, gas exchange values in the leaves of control plants treated with GABA application were significantly increased (p ≤ 0.05) as compared to those without GABA application. By increasing the concentration of GABA, the highest values of Pn, Gm, Tr, and gs were observed in control and salinity-stressed plants. Pn, Tr, and Ci increased significantly, with the largest extent by 6.42 µmol CO_2_ m^− 2^ s^− 1^, 3.96 mmol H_2_O m^− 2^ s^− 1^, and 287.75 µmol CO_2_ mol^− 1^, respectively, upon exogenous GABA treatment (40 mM) + control conditions in the ‘Rabab’ plants. Moreover, the ‘Atabaki’ plants treated with the foliar application (40 mM GABA) improved Pn, Tr, and Ci, by 6.33 µmol CO_2_ m^− 2^ s^− 1^, 3.73 mmol H_2_O m^− 2^ s^− 1^, and 275.5 µmol CO2 mol^− 1^, respectively, with the largest extent under control conditions. Though there were no significant changes between the gs in the ‘Atabaki’ cultivar under drought stress with or without GABA treatment (p ≤ 0.05). Among the gas exchange parameters, Ls was decreased by increasing the concentration of GABA application, and the least extent of Ls was observed under control conditions and 40 mM GABA with increases of 0.21% in the ‘Rabab’ cultivar (Fig. 4). In the leaf gas exchange parameters, small differences were observed between the two experimented cultivars under all the treatments.

### Sugar contents (total soluble carbohydrate, glucose, fructose, and sucrose content) and starch regulated in response to exogenous GABA at drought-salinity stress

In general, the application of GABA increased sugar contents under non-stressed conditions. In comparison with the control treatment, remarkably, exogenous application of GABA, especially in 40 mM concentration, significantly increased TSC, glucose, and fructose contents in the leaves of pomegranate plants under stress conditions (p ≤ 0.05, Table 1). However, the increase in TSC content by exposure to GABA treatment was greater under drought and the interaction between these two stresses than salinity stress. The highest TSC content in the leaves under drought-salinity stress conditions was observed, at 18.6 mg g^− 1^ DW in the ‘Rabab’ cultivar and 81.34 mg g^− 1^ DW in the ‘Atabaki’ cultivar (p ≤ 0.05). Moreover, significant increments of glucose and fructose contents were recorded for the ‘Rabab’ cultivar at 30.13 mg g^− 1^ DW and 30.62 mg g^− 1^ DW, respectively, with 40 mM GABA foliar application under drought and salinity combination and for ‘Atabaki’ cultivar at 30.88 mg g^− 1^ DW under water deficit stress and 40 mM GABA treatment and 33.10 mg g^− 1^ DW with 40 mM GABA foliar application under drought and salinity stress combined (Table 1).

**Table 1:** The effect of GABA concentrations and cultivars subjected to salinity-drought stress on the sugar contents of pomegranate plants

Cultivar	Stress	GABA (mM)	Total soluble carbohydrate (mg g^− 1^ DW)	Starch (mg g^− 1^ DW)	Glucose (mg g^− 1^ DW)	Fructose (mg g^− 1^ DW)	Sucrose (mg g^− 1^ DW)
‘Atabaki’	C	0	19.02^o^ ± 3.201	16.10^ m − o^±1.169	16.08^q^ ± 0.777	10.37^p^ ± 0.863	5.59^f − h^±0.608
		10	24.17^n^ ± 2.603	16.72^k − o^±5.798	16.95^pq^ ± 0.317	14.26^mn^ ± 1.244	6.57^d^ ± 0.512
		20	26.92^n^ ± 3.195	17.09^j − n^±5.578	17.62^op^ ± 0.640	15.33^ m^ ± 0.689	7.69^c^ ± 1.209
		40	37.60^i − l^±3.065	33.37^a^ ± 3.851	19.09^mn^ ± 0.929	15.01^ m^ ± 1.054	7.81^c^ ± 0.345
	D	0	45.00^ g − h^±4.665	12.26^o − p^±2.816	22.80^k^ ± 0.141	18.70^kl^ ± 0.374	2.67^o^ ± 0.280
		10	49.62^ fg^ ± 0.892	16.27^ L − o^±5.302	27.45^d^ ± 0.657	19.84^i − k^±0.562	3.45^mn^ ± 0.406
		20	65.73^b^ ± 6.519	29.91^ab^ ± 2.833	29.11^c^ ± 0.825	21.74^ g^ ± 1.225	3.88^k − m^±0.175
		40	49.21^ g^ ± 4.778	24.92^d − g^±5.453	30.88^a^ ± 0.382	25.33^de^ ± 0.914	5.33^ g − i^±0.241
	S	0	28.59^mn^ ± 1.032	18.76^ h − m^±4.240	22.81^k^ ± 0.298	18.84^j − l^±0.414	3.09^no^ ± 0.300
		10	35.73^kl^ ± 3.836	18.04^ h − m^±2.062	23.46^i − k^±0.807	21.72^ g^ ± 1.820	4.29^jk^ ± 0.257
		20	38.13^i − k^±0.978	22.55^d − h^±1.132	23.89^ h − j^±0.542	23.60^f^ ± 0.429	5.41^f − i^±0.403
		40	39.08^i − k^±2.410	25.38^c − f^±0.756	25.85^ef^ ± 1.174	24.85^d − f^±0.604	5.63^f − h^±0.378
	D×S	0	37.04^jl^ ± 2.729	10.26^p^ ± 2.859	23.96^ g − i^±0.571	21.82^ g^ ± 2.202	2.52^o^ ± 0.331
		10	41.02^ h − j^±3.940	17.93^i − m^±2.493	24.59^gh^ ± 0.729	28.76^c^ ± 2.041	3.80^k − m^±0.645
		20	60.38^ cd^ ± 4.552	19.04^ h − m^±1.565	26.97^d^ ± 1.115	28.06^c^ ± 0.740	4.12^kl^ ± 0.289
		40	58.42^de^ ± 2.366	20.75^ g − l^±1.046	30.40^ab^ ± 1.067	33.10^a^ ± 0.646	4.99^hi^ ± 0.404
‘Rabab’	C	0	16.43^o^ ± 6.296	18.83^ h − m^±3.899	16.29^q^ ± 0.341	6.82^r^ ± 0.656	6.48^de^ ± 0.538
		10	32.58^ lm^ ± 2.793	21.73^e − i^±2.581	17.74^op^ ± 0.312	8.39^q^ ± 0.740	7.53^c^ ± 0.284
		20	26.77^n^ ± 5.343	26.52^b − d^±1.366	18.38^no^ ± 0.258	11.86^o^ ± 1.663	8.53^b^ ± 0.202
		40	37.71^i − k^±1.683	29.57^a − c^±1.637	19.40^ m^ ± 0.345	13.30^no^ ± 0.582	9.44^a^ ± 0.432
	D	0	39.25^i − k^±1.047	13.38^np^ ± 1.296	23.20^i − k^±0.394	17.84^ L^ ± 0.563	3.54^ L − n^±0.675
		10	40.95^ h − j^±1.900	17.99^i − m^±1.201	24.67^gh^ ± 0.919	18.93^j − l^±0.522	5.14^hi^ ± 0.852
		20	42.31^ h − i^±4.397	21.62^e − i^±5.533	27.68^d^ ± 0.622	20.23^ h − j^±0.316	5.59^f − h^±0.542
		40	54.76^e^ ± 4.308	25.24^c − g^±4.409	29.02^c^ ± 1.289	21.70^gh^ ± 0.927	5.85^e − g^±0.508
	S	0	38.55^i − k^±1.139	12.32^n − p^±2.936	21.62^ L^ ± 0.566	17.69^ L^ ± 0.499	3.60^ L − n^±0.295
		10	37.83^i − k^±1.494	20.99^f − k^±3.614	22.95^jk^ ± 0.749	19.48^jk^ ± 0.647	4.17^kl^ ± 0.300
		20	40.83^ h − j^±1.302	21.40^f − j^±2.237	26.73^de^ ± 0.821	21.87^ g^ ± 1.686	5.38^ g − i^±0.485
		40	41.68^ h − j^±1.530	25.95^b − e^±3.379	28.73^c^ ± 0.406	23.90^ef^ ± 1.282	6.05^d − f^±0.264
	D×S	0	54.29^ef^ ± 1.932	9.76^p^ ± 2.616	24.88^gf^ ± 0.515	21.15^ g − i^±0.534	2.59^o^ ± 0.408
		10	63.65^bc^ ± 4.137	16.41^ L − o^±1.707	25.80^ef^ ± 0.310	23.72^f^ ± 1.211	4.17^kl^ ± 0.164
		20	55.64^de^ ± 5.369	19.63^ h − m^±2.053	29.43^bc^ ± 0.487	26.17^d^ ± 0.660	4.28^jk^ ± 0.081
		40	81.34^a^ ± 6.207	19.95^ h − m^±1.984	30.13^ab^ ± 1.014	30.62^b^ ± 1.225	4.90^ij^ ± 0.132

Mean ± standard deviation (SD). Control (C), drought (D), salinity (S), drought and salinity (D×S).

In both cultivars, starch content significantly decreased under stress; however, compared with the starch degradation in plants under stress without GABA treatment, GABA treatment significantly increased the starch content (p ≤ 0.05). However, slight increases in starch content in pomegranate leaves under the combination of drought and salinity stress were observed as compared to drought and salinity stress alone. Among GABA-treated plants, the maximum increase of 33.37 mg g^− 1^ DW was observed in ‘Atabaki’ plants when subjected to control + 40 mM GABA treatment. Similarly, for sucrose content, a significant reduction was recorded under stress treatments for both cultivars compared with the control treatment. In both control and stressed plants, treatments with GABA had higher sucrose content than treatments without GABA. Meanwhile, the treatment that combined stress with GABA revealed the opposite pattern compared with only stress treatment. Furthermore, the 40 mM GABA + control plants showed the remarkably highest sucrose content of 29.57 mg g^− 1^ DW in the ‘Rabab’ cultivar and 33.37 mg g^− 1^ DW in the ‘Atabaki’ cultivar (Table 1). Under normal and stressful conditions, TSC, starch, glucose, fructose, and sucrose contents in the leaves of untreated and GABA-treated plants (two cultivars) were relatively similar.

### Nutrient elements change in response to drought-salinity stress alone and with GABA treatment

**Fig. 5:**
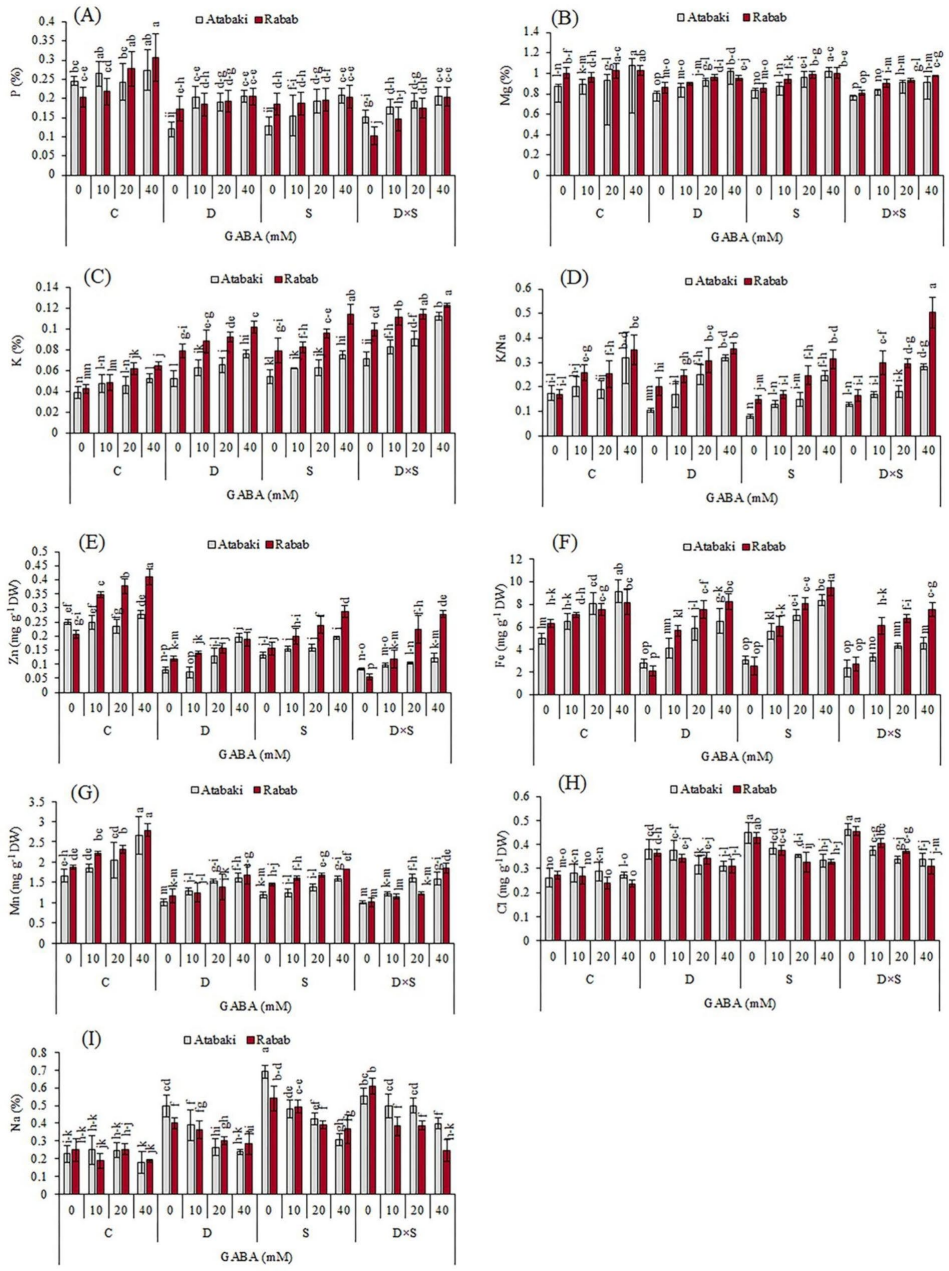
Effect of exogenous GABA on nutrient elements in pomegranate plants subjected to drought and salinity stresses. (A) phosphorus (P), (B) magnesium (Mg), (C) potassium (K), (D) potassium/sodium (K/Na), (E) zinc (Zn), (F) iron (Fe), (G) manganese Mn, (H) chloride (Cl) and (I) sodium (Na). Control (C), drought (D), salinity (S), and drought-salinity (D×S). Vertical columns indicate Mean ± standard deviation (SD)

The nutrient element changes for all treatments are presented in Fig. 5. The concentration of most nutrient elements (P, Mg, K/Na, Zn, Fe, and Mn) in the leaves was remarkably higher under control treatment than under stress treatment, while the concentration of K, Na, and Cl increased under stress treatment (Fig. 5). By contrast, increasing the concentration of exogenous GABA application (up to 40 mM) had a profound effect on nutrient elements, increasing the concentration of P, Mg, K, K/Na, Zn, Fe, and Mn under both control and stress conditions, across both cultivars. GABA application (in different concentrations) also had a significant effect on the concentration of Na and Cl in stress-treated plants and decreased the concentration of Na and Cl. Under GABA treatment, the concentrations of P, Mg, Zn, and Mn in control plants showed a significant increase when compared to pomegranate plants under stress treatments. So that under the control treatment, the 40 mM GABA treatment significantly increased P, Mg, Zn, and Mn contents in the leaves by 0.31%, 1.03%, 0.41 mg g^− 1^ DW, and 2.79 mg g^− 1^ DW in the ‘Rabab’ cultivar, and by 0.27%, 1.07%, 0.28 mg g^− 1^ DW, and 2.65 mg g^− 1^ DW in the ‘Atabaki’ cultivar, respectively (p ≤ 0.05, Fig. 5). K concentration was significantly increased in GABA-treated as well as drought- and salinity-stressed plants with respect to control. The plants treated with 40 mM GABA under the combination of drought and salinity stress exhibited an obvious concentration of K, of 12% and 11% in ‘Rabab’ and ‘Atabaki’, respectively, compared with other treatments. Additionally, the concentration of K/Na in ‘Rabab’ plants under the combination of drought and salinity stress and 40 mM GABA showed the most significant increase of 0.50 relative to the ‘Atabaki’ cultivar and other treatments (p ≤ 0.05). The 40 mM GABA treatment significantly increased the Fe concentration, with an increase of 9.44 mg g^− 1^ DW under salinity stress for the ‘Rabab’ cultivar and 9.16 mg g^− 1^ DW under control conditions for the ‘Atabaki’ cultivar. The Na concentration was significantly increased under stress conditions, attaining maximal values in the ‘Rabab’ cultivar by 0.61% when exposed to the combination of drought and salinity stress and by 0.69% in the ‘Atabaki’ when exposed to salinity stress. The concentration of Cl was also increased in stress treatments compared to controls. The highest Cl concentration was obtained in plants exposed to salinity stress or a combination of drought and salinity stress. The Cl concentration was significantly decreased after the application of GABA in plants under stress treatments (p ≤ 0.05). Under normal water conditions, there were no significant differences between plants treated with or without GABA in Na and Cl concentrations. Changes in nutrient elements were similar among both cultivars; however, increases in K concentration in ‘Rabab’ leaves under stress treatments were higher than those in ‘Atabaki’ leaves (Fig. 5).

### Principal component and heatmap cluster analysis

**Fig. 6:**
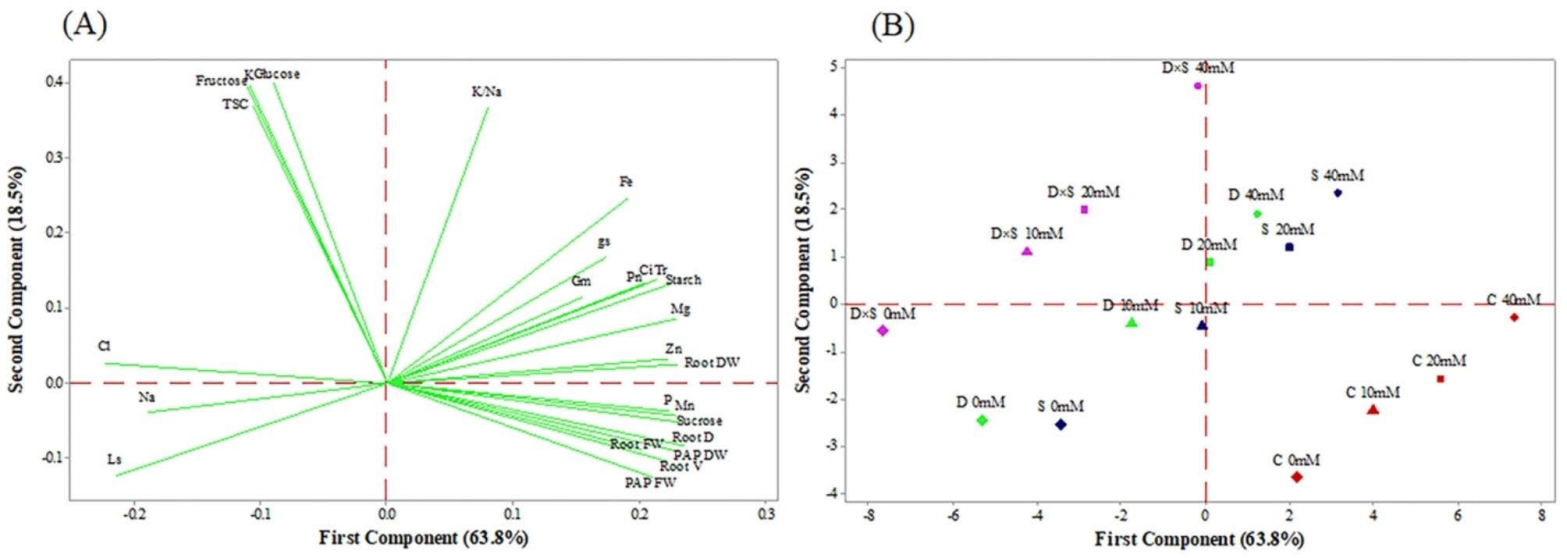
The principal component analysis score plot of (A) morpho-physicochemical parameters, (B) control and GABA-treated samples in the ‘Rabab’ cultivar. Stomatal limitation (Ls), mesophyll conductance (G_m_), net photosynthetic rate (Pn), stomatal conductance of water vapour (gs), transpiration rate (Tr), intercellular carbon dioxide concentration (Ci), plant aerial part of fresh weight (PAP FW), plant aerial part dry weight (PAP DW), root fresh weight (Root FW), root dry weight (Root DW), root diameter (Root D), root volume (Root V), chloride (Cl), magnesium (Mg), sodium (Na), potassium (K), phosphorus (P), manganese (Mn), zinc (Zn), and iron (Fe), potassium/sodium (K/Na). *Note*: Red, blue, green and purple dots represent control (C), salinity (S), drought (D) and drought and salinity combined stress (D×S), respectively. Also, 0 mM (diamond), 10 mM (triangle), 20 mM (square), and 40 mM (circle) represent different concentrations of GABA treatment

**Fig. 7:**
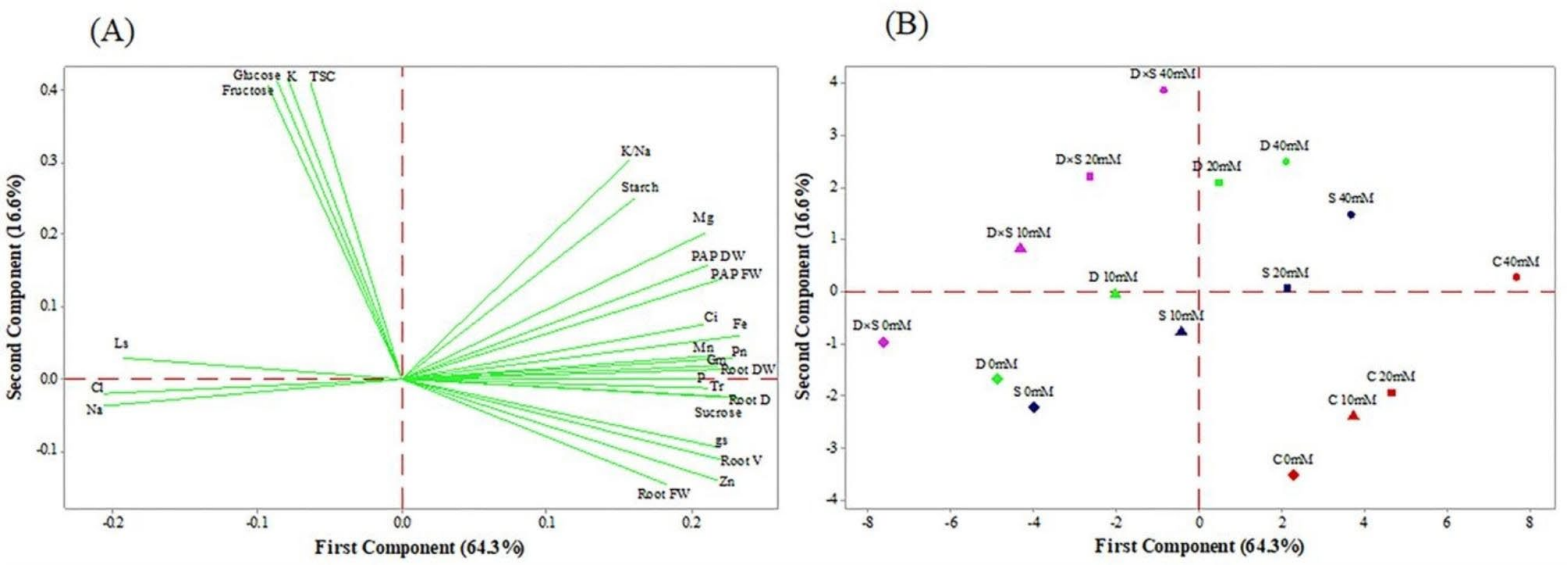
The principal component analysis score plot of (A) morpho-physicochemical parameters, (B) control and GABA-treated samples in the ‘Atabaki’ cultivar. Stomatal limitation (Ls), mesophyll conductance (G_m_), net photosynthetic rate (Pn), stomatal conductance of water vapour (gs), transpiration rate (Tr), intercellular carbon dioxide concentration (Ci), plant aerial part fresh weight (PAP FW), plant aerial part dry weight (PAP DW), root fresh weight (Root FW), root dry weight (Root DW), root diameter (Root D), root volume (Root V), chloride (Cl), magnesium (Mg), sodium (Na), potassium (K), phosphorus (P), manganese (Mn), zinc (Zn), and iron (Fe), potassium/sodium (K/Na). *Note*: Red, blue, green and purple dots represent control (C), salinity (S), drought (D) and drought and salinity combined stress (D×S), respectively. Also, 0 mM (diamond), 10 mM (triangle), 20 mM (square), and 40 mM (circle) represent different concentrations of GABA treatment

To visualize sample grouping, a principal component analysis (PCA) based on the physiochemical and biochemical parameters was performed separately for both cultivars to better analyze the relationships among the studied traits and inspect how they were associated with each cultivar (Figs. 6 and 7). The first two PCs accounted for 82.3% and 80.9% of the total variance in the ‘Rabab’ and ‘Atabaki’ cultivars, respectively. The first PC axis, which explained 63.8% and 64.3%, respectively, of the variation in ‘Rabab’ and ‘Atabaki’ cultivars, was primarily related to sucrose, starch, P, Fe, Mg, Mn, Zn, K/Na, root fresh and dry weight, plant fresh and dry weight, Pn, Gm, Tr, gs, Ci, root diameter and volume. The second axis, which explained 18.5% of the variation in the ‘Rabab’ cultivar, was mainly associated with glucose, fructose, TSC, starch, Fe, Mg, Zn, Cl, K/Na, K, root dry weight, Pn, Gm, Ci, Tr, and gs. Also, the second axis, which explained 16.6% of the variation in the ‘Atabaki’ cultivar, was driven mainly by glucose, fructose, TSC, starch, Fe, Mn, Mg, K/Na, K, root dry weight, plant fresh and dry weight, Pn, Gm, Ci, and Ls. In general, relationships among traits along the first and second axes in the two studied cultivars showed a similar pattern (Fig. 6A). With respect to Figs. 6B and 7B, in both cultivars, the clustering analyses indicated that non-stressed plants, especially those exposed to GABA treatment, were clustered separately (separation along PC1) from the stress treatments due to the higher variables related to growth and physio-biochemical traits. The three studied stress conditions were positively correlated with Cl, Na, and Ls, whereas under both drought and salinity stress conditions, 20 and 40 mM GABA treatments were positively correlated with variables related to Gm, gs, Fe, and K/Na values in ‘Rabab’ cultivar. In the ‘Atabaki’ cultivar, the Cl, Na, and Ls values were associated with drought salinity and drought and salinity combined stress conditions with 10 mM GABA treatment. As for K, TSC, glucose, and fructose, they shared the same cluster in both cultivars that were exposed to drought and salinity combined stress conditions with 20 mM GABA treatment.

**Fig. 8:**
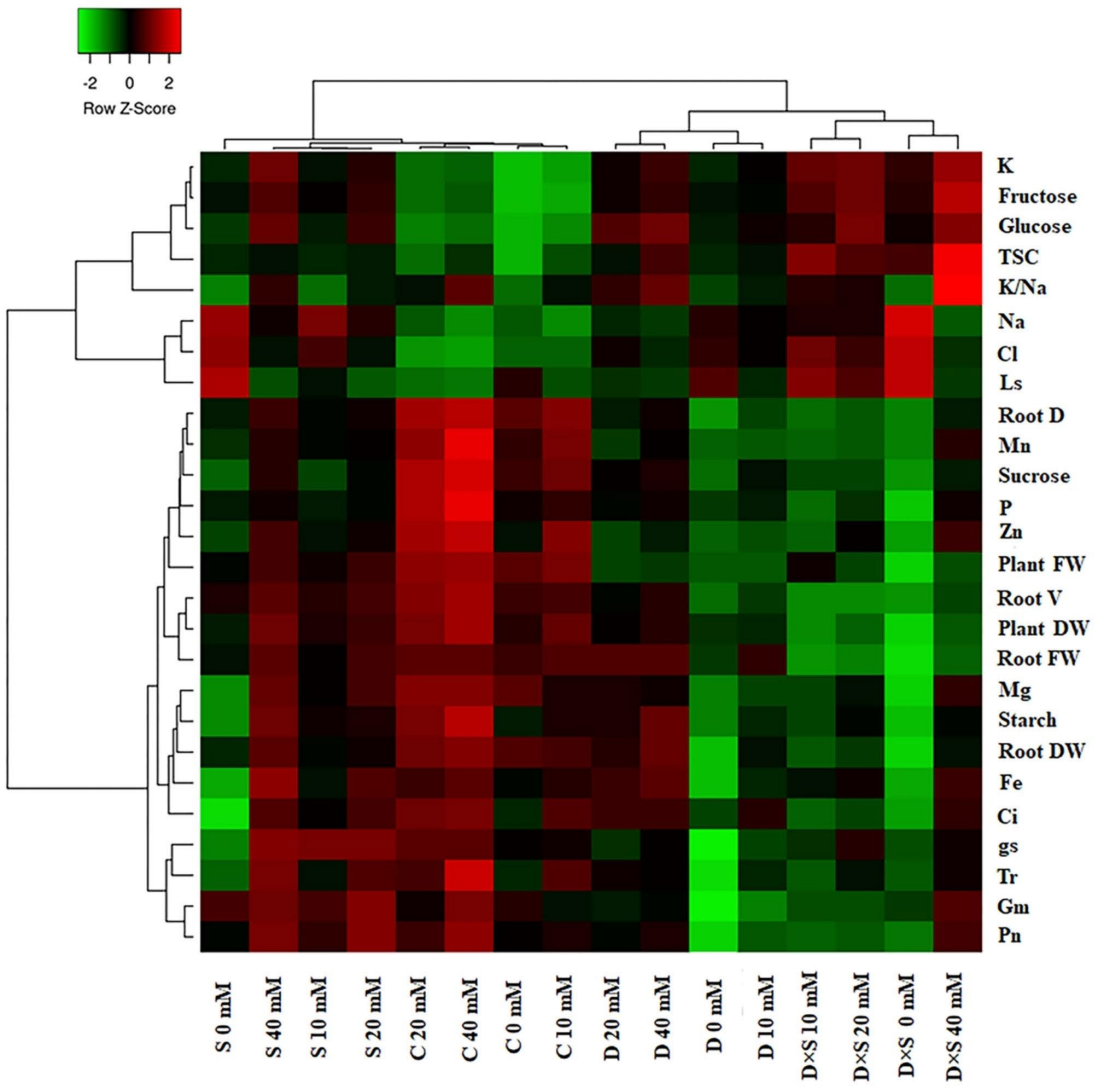
Hierarchical clustering and heatmap diagram of all measured plant parameters in response to exogenous GABA application and drought and salinity stress in the ‘Rabab’ cultivar. Stomatal limitation (Ls), mesophyll conductance (G_m_), net photosynthetic rate (Pn), stomatal conductance of water vapour (gs), transpiration rate (Tr), intercellular carbon dioxide concentration (Ci), plant aerial part fresh weight (PAP FW), plant aerial part dry weight (PAP DW), root fresh weight (Root FW), root dry weight (Root DW), root diameter (Root D), root volume (Root V), chloride (Cl), magnesium (Mg), sodium (Na), potassium (K), phosphorus (P), manganese (Mn), zinc (Zn), and iron (Fe), potassium/sodium (K/Na). Control (C), drought (D), salinity (S), and drought-salinity (D×S)

**Fig. 9:**
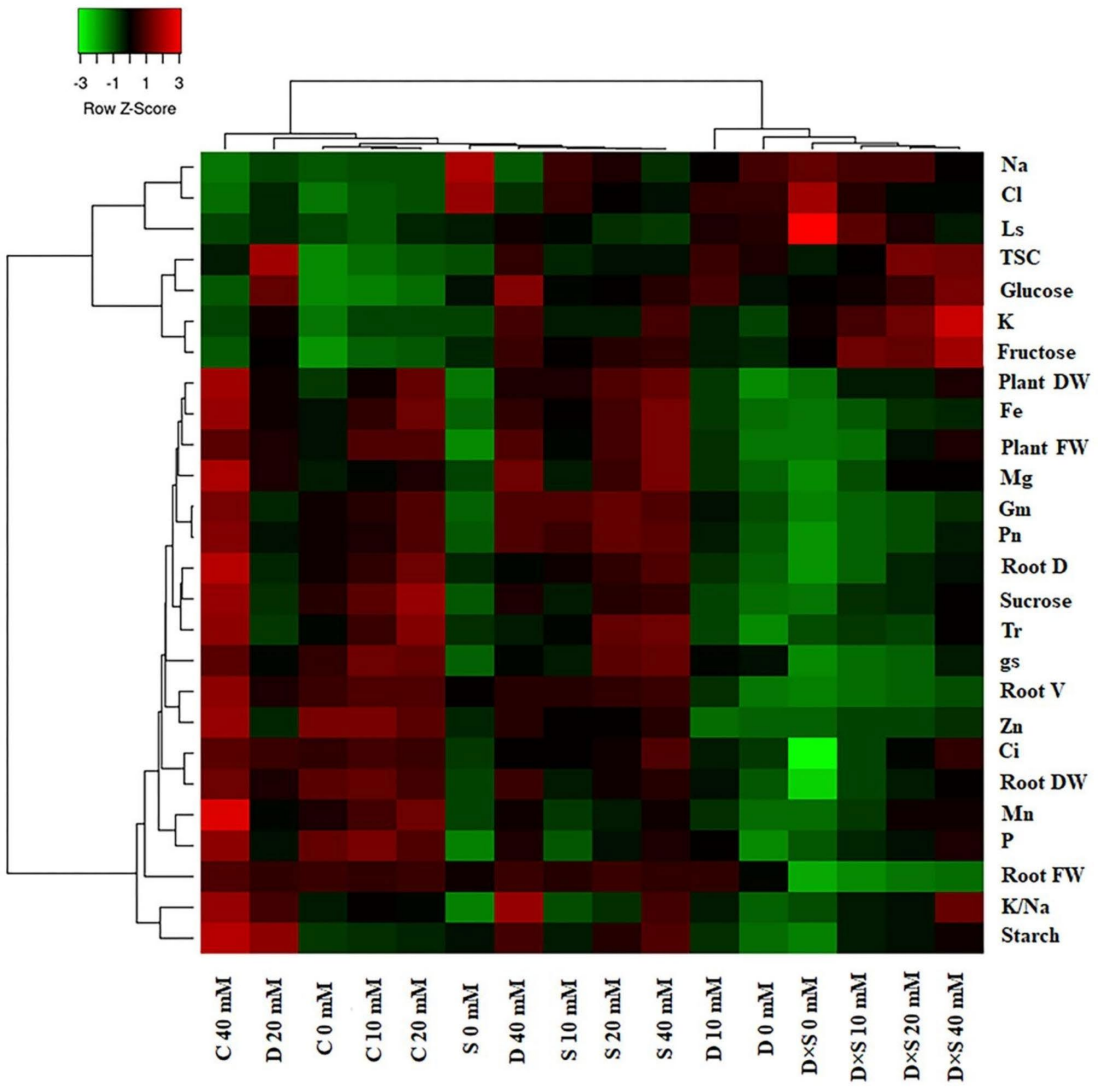
Hierarchical clustering and heatmap diagram of all measured plant parameters in response to exogenous GABA application and drought and salinity stress in the ‘Atabaki’ cultivar. Stomatal limitation (Ls), mesophyll conductance (G_m_), net photosynthetic rate (Pn), stomatal conductance of water vapour (gs), transpiration rate (Tr), intercellular carbon dioxide concentration (Ci), plant aerial part fresh weight (PAP FW), plant aerial part dry weight (PAP DW), root fresh weight (Root FW), root dry weight (Root DW), root diameter (Root D), root volume (Root V), chloride (Cl), magnesium (Mg), sodium (Na), potassium (K), phosphorus (P), manganese (Mn), zinc (Zn), and iron (Fe), potassium/sodium (K/Na). Control (C), drought (D), salinity (S), and drought-salinity (D×S)

Correlation analysis and hierarchical clustering were conducted on the whole experimental data set to investigate the relationships between measured morpho-physicochemical parameters and tested treatment factors. Hierarchical clustering and heatmap diagram have been depicted in Figs. 8 and 9. Overall, most of the measured parameters in both cultivars had the lowest correlation under stress treatments, especially drought-salt stress without GABA application, compared to control plants and stressed plants with GABA treatment. However, K, glucose, fructose, and TSC exhibited the most changes under stress treatments with increasing concentrations of GABA treatment, in comparison to non-stress treatment, in both cultivars. Moreover, Ls, Na, and Cl demonstrated a high correlation under stress conditions, but their correlation decreased under the 40 mM GABA concentration (Figs. 8 and 9). The hierarchical cluster analysis distinctly grouped stress-treated and control plants with or without GABA application, which exemplifies that drought + 0 and 10 mM GABA and drought-salinity + all concentrations of GABA were grouped together and the other treatments were clustered into a separate group in the ‘Atabaki’ cultivar (Fig. 8). In the ‘Rabab’ cultivar, dendrogram related to the treatments revealed two main groups: drought and drought-salinity stresses with or without GABA treatment were grouped together and the other treatments were clustered into a separate group (Fig. 9). According to hierarchical clustering results for the ‘Atabaki’ cultivar, all measured parameters were grouped into four clusters. At the top of the heat map, the first cluster of measured parameters contains negative correlations (i.e., Na, Cl, and Ls), while the second cluster at the bottom of the heat map mostly groups positive and negative correlations (i.e., TSC, glucose, fructose, and K content). The third cluster is the mixed cluster with nutrient elements, morphological traits, sucrose, and photosynthetic characteristics. The fourth cluster corresponded to K/Na and starch contents (Fig. 8). In the ‘Rabab’ cultivar, the first cluster contains K, K/Na, fructose, glucose, and TSC; then at the bottom of the heat map, the second cluster consists of the Na, Cl, and Ls levels (all parameter values were low). The third cluster is mainly for the parameters of root diameter, root volume, sucrose, Mn, P, Zn, Mg, Fe, Ci, starch, and fresh and dry weight of plant and root. The fourth cluster clearly revealed the Gm, Tr, gs, and Pn (Fig. 9).

## Discussion

### Morphological characteristics in response to drought and salinity stress alone and with GABA treatment

In the present study, drought- or salinity-caused oxidative damage substantially decreased the growth indices of two pomegranate cultivars, i.e., ‘Rabab’ and ‘Atabaki’, in terms of the plant aerial part of fresh and dry weight, root- fresh and dry weight, diameter and volume of the root. Interestingly, simultaneous exposure to both salinity and drought stress had exerted even more hazards to plant growth than either stress alone. The root system plays a vital role in the absorption of nutrients and water from the soil. In the face of drought and salinity, the root system experiences diminished turgor pressure, resulting in a suppression of cell enlargement, cell division, and overall growth. Consequently, a smaller root system is an adverse outcome of these stresses [26]. Additionally, the observed decline in root biomass under stress conditions can be attributed to the inefficiency of root respiration, which ultimately leads to reduced nutrient and water absorption by the roots [39]. Such growth inhibition by the simulated drought and salinity stress has previously been reported on pomegranate [3, 40] as well as other plant species [25, 41]. However, GABA application had a significant positive impact on pomegranate growth, promoting the diameter and distribution of roots under stress and non-stress conditions. Moreover, GABA application had an even greater effect under non-stress conditions. The findings of our study suggest that root formation is regulated by several factors, including hormones and C/N metabolism, which are linked to the GABA shunt pathway [42, 43]. In addition to its role as a source of C and N, our research supports the idea that GABA also functions as a signaling molecule. It appears to have a specific effect on the uptake and assimilation of nitrate, which improves N availability and ultimately promotes plant growth. Thus, the observed increase in root volume and diameter indicates a distinct influence of GABA on root growth, rather than solely acting as a N source. GABA triggers the synthesis of abscisic acid (ABA), primarily in the roots. ABA is involved in modifying root architecture by promoting root elongation. This process allows plants to access deeper water sources in the soil during periods of water deficit. Additionally, ABA enhances the synthesis of osmoprotectants, which help plants withstand harsh environmental conditions [4]. Recent research has revealed that GABA can modify the activity of ALUMINUM-ACTIVATED MALATE TRANSPORTERS (ALMTs), which are implicated in aluminum tolerance, root growth, pollen tube growth, and stomatal pore regulation [44]. These findings provide valuable insights into the mechanism by which GABA acts as a membrane potential-based signal, connecting plant metabolic status with various physiological processes [45]. Our current findings are also in accordance with previous research that has suggested that GABA treatment can contribute to promote plant growth, which is most closely associated with the photosynthesis rate. This is because a reduced rate of photosynthesis leads to a decrease in the biosynthesis of carbohydrates that are used for plant growth [42]. Furthermore, such increases in root growth under stress conditions in GABA-treated plants might be beneficial to the uptake of moisture and nutrient elements [20]. Previous studies have reported that GABA application mitigates the inhibitory effects of stress on root growth in rice plants [46], increases the fresh weight and root length of barley under toxicity conditions [47], and maintains higher shoot dry weight, fresh weight, and leaf area in snap bean seedlings exposed to drought stress [41]. Consistent with these findings, our previous report also demonstrated that GABA-treated pomegranate plants exhibited significant increases in vegetative structures such as plant height, crown diameter, leaf area, leaf length, and leaf width during periods of drought and salinity stress [26]. This notable morphology response can be attributed to the stimulating effects of GABA on both cell division and elongation processes, ultimately leading to a significant increase in plant biomass.

### Effects of exogenous GABA and drought-salinity stress on photosynthesis and gas exchange parameters

Photosynthesis is a crucial process for plant growth and development, as it forms the basis for the accumulation of dry matter [31]. Our study revealed that drought and NaCl stress inhibit photosynthetic capacity by decreasing Ci and gs, which were accompanied by continuously declining Pn, Gm, and Tr; however, Ls demonstrated an increase. This finding may be associated with the reduction of intercellular carbon dioxide concentration in the cellular spaces of the leaf, possibly inhibiting carbon uptake and disturbing carbon balance, which ultimately inhibits plant growth [48]. However, drought and salinity stress damage the physiological metabolism and photosynthesis activity of plants and reduce crop production [49]. Nevertheless, our results revealed that GABA-treated plants maintained high photosynthesis capacity due to their related characteristics, including Pn, Tr, Gm, Ci, and gs, and decreased Ls levels in response to drought and salinity conditions. Similarly to our results, previous studies have reported that GABA can protect plants by enhancing the efficiency of their photosynthetic assimilation capacity under environmentally stressful conditions [16, 25]. The finding of Xiang et al. [31] indicated that GABA-regulated salinity-alkalinity tolerance may be associated with increasing the levels of ABA in plants and as a result, help plants maintain the rate of Pn under the decreased CO_2_ concentrations resulting from stomatal closure by regulating stomatal aperture (promoting stomatal opening). We interpret that drought and salinity stress may also block the photosynthetic electron transport chain, which in turn damages the photosynthetic apparatus and exogenous GABA alleviates these effects [31]. The GABA shunt pathway through succinate production in plants, an intermediate of the tricarboxylic acid (TCA) reaction, provides a substrate for both the TCA cycle and the electron transport chain, so succinic acid maintains the carbon-nitrogen cycle in plants [50]. This research was confirmed by Xiang et al. [31], who found that the direct effect of GABA treatment increased the amount of photosynthesis capacity in melon plants. It was also observed that GABA application resulted in improvements in photosynthetic efficiency in waterlogged treated maize seedlings [13], which had been mostly caused by maintaining cell turgor through the cytosolic accumulation of osmolytes [22], alleviation of chlorophyll degradation and preservation of the ultrastructure of functional leaves [13]. Similarly, Wang et al. [51] found that the application of GABA + salinity stress promoted higher Pn and gs than maize that received NaCl alone.

### Sugar contents (total soluble carbohydrate, glucose, fructose, and sucrose content) and starch regulated in response to exogenous GABA at drought-salinity stress

Our findings revealed a significant enhancement in the catabolism of starch and the increase of TSC, glucose, fructose, and sucrose contents in response to both drought and salinity stress. Huang et al. [52] reported that TSC reserves tend to decline under short-term drought stress but increase during long-term drought simulation experiments. However, when GABA + stress treatments were applied, these parameters exhibited even greater increases. This suggests that GABA-controlled resistance mechanisms against drought and salinity stresses may involve the accumulation of soluble sugars and starch. Moreover, the exogenous GABA supplementation also enhanced TSC content under non-stress conditions. Our findings aligns with previous research indicating that exogenous GABA supplementation can enhance the accumulation of soluble carbohydrates under drought stress in various plant species, such as black cumin (*Nigella sativa* L.) [20] and strawberry plants [53]. Similarly, GABA has been shown to regulate salinity tolerance in maize corn by increasing the contents of soluble carbohydrates and proline in maize leaves [51]. Similar results were observed in tomato plants, where exogenous GABA clearly enhanced starch content and potentially acted as a reserve to induce sucrose formation (osmolyte accumulation) during chilling stress [20]. These findings suggest a close relationship between GABA and C/N metabolism. Additionally, the increased sugar contents may indicate their role as signaling molecules, interacting with hormone pathways and regulating root growth and development [54]. The positive effect of GABA on osmotic adjustment and turgor pressure maintenance in plants highlights its ability to modulate the flow of photoassimilates. Therefore, the enhanced carbohydrate accumulation induced by GABA in pomegranate plants may contribute to improved osmotic adjustment, sugar transport, enhanced photosynthesis (Pn), and efficient water absorption, which are crucial functions for plants to tolerate water deficit and salinity [20, 51]. Moreover, disaccharides such as sucrose and trehalose, as well as hexoses like glucose and fructose, serve as important osmolytes in plant cells. These osmolytes not only help maintain cellular balance but also act as signaling molecules, triggering the expression of various genes, including those involved in defense mechanisms [12]. Sucrose serves as the primary photosynthetic product in higher plants and is responsible for long-distance transport through the phloem [55]. The GABA shunts also play a significant role in providing energy and carbon structures during periods of stress. Yu et al. [14] have reported that exogenous GABA significantly promotes the contents of sucrose and glucose, potentially by maintaining a normal TCA cycle. Similar findings were reported by Li et al. [17], who indicated that the carbon skeletons of exogenous GABA entering the TCA cycle facilitate carbon assimilation, leading to the accumulation of sucrose and glucose, thereby improving the nutritional content of creeping bentgrass (*Agrostis stolonifera*).

### Nutrient elements change in response to drought-salinity stress alone and with GABA treatment

Mineral nutrients, particularly macronutrients, play a critical role in the plant structure and physiological functions of plants. In fact, the deficiency of these mineral nutrients can have a significant influence on plant growth and development. Micronutrients are also equally critical as they are components of several enzymes that directly or indirectly affect the regulating enzyme-catalyzed reactions and metabolites involved in plant responses to adverse environmental conditions [48, 56, 57]. There have been multiple reports indicating that several factors, such as external environmental conditions and the plant itself, have a substantial influence on nutrient absorption [29]. When examining the effects of stress treatments on pomegranate plants, it was found that a number of essential nutrients, including Zn, Mn, Fe, K/Na, and Mg, were decreased. Conversely, K, Cl, and Na levels increased significantly. Hence, this increase in Na, Na/K, and Cl in plants has been implicated in the generation of ROS. ROS, in turn, disrupt stomatal closure, diminish photosynthetic activity, and facilitate lipid peroxidation in the plant [58]. Consequently, the observed growth phenotype of pomegranate cultivars could be partly attributed to the reduced uptake of mineral nutrients. Furthermore, the increase of the K content in stress-treated plants was mainly attributed to the increased uptake of K facilitated by Na. In line with our findings, similar studies have reported elevated K content in pomegranate [3] and oleander (*Nerium oleander* L.) leaves [59] in response to salinity stress. It has been suggested in numerous studies that the increment in nutrient elements, particularly K, is closely associated to the plant’s ability to survive under stressful conditions [56]. Additionally, K plays a crucial role in enhancing photosynthetic capacity by elevating the concentration of photosynthetic pigments and promoting photosynthate production through stomatal opening [58]. However, the exogenous supplementation of GABA, particularly at a high concentration of 40 mM, resulted in increased nutrient element absorption in both non-stressed and stressed conditions compared to untreated plants. GABA exhibited a greater impact on the K and K/Na contents in drought-salinity treated plants compared to those subjected to drought or salinity alone, highlighting the vital role of GABA in the osmotic adjustment of stressed plants. Recent reports have demonstrated that exogenous GABA significantly promotes K, Ca, Mg, and Zn uptake in melon plants (*Cucumis melo* L.) under low oxygen stress [60], enhances K uptake and K/Na ratio in salt-stressed maize seedlings [61], and improves K and Mg uptake in *Monoraphidium* sp. under cadmium stress [62]. Moreover, the role of exogenous supply of GABA by increscent absorption of N, P, and K in loquat seedlings [29] and Na, Mn, Zn, and Fe contents in fragrant rice [39] has also been confirmed under normal conditions. Our study has, for the first time, demonstrated the involvement of GABA in protecting mineral nutrients uptake from drought and salinity stress in pomegranate plants. In terms of nutrient uptake, GABA may modulate the metabolism of free amino acids through the regulation of ALMT, which in turn mediates nitrogen (N) metabolism and uptake in pomegranate [63, 64]. These results support the hypothesis that one of the functions of GABA in plants is to regulate ion transport and enhance mineral absorption capacity [65], thereby effectively mitigating oxidative damage caused by drought or salinity while promoting plant growth. Another study by Yang et al. [29] found that succinate production through GABA metabolism (the shunt pathway of GABA) not only helps maintain the normal TCA cycle but also increases organic acid content in plant roots and stems while reducing cellular acidity. As a result, it effectively regulates the absorption of mineral elements by plants [29]. Whether the interaction between GABA and ion transporters is direct or indirect requires further investigation. This study, for the first time, highlights the positive effects of GABA in protecting pomegranate plants against oxidative damage induced by various stresses (drought and salinity stress) through the accumulation of sugar contents and mineral nutrients, which are crucial for improving photosynthetic capacity and growth characteristics. Nonetheless, it is crucial to note that further research is still needed to fully decipher the involvement of GABA-mediated regulations in plant growth, as well as the intricate molecular and genetic mechanisms associated with biochemical mechanisms.

## Conclusion

According to the results of this study, both drought and salinity stress have similar negative effects on pomegranate plants, including reduced growth, disrupted photosynthesis, and impaired mineral nutrient uptake. However, the foliar application of GABA, particularly at higher concentrations (40 mM), effectively alleviated oxidative damage caused by drought, salinity, and their interactions through the accumulation of sugar contents (TSC, glucose, fructose, and sucrose), enhancement of photosynthetic capacity, and promotion of mineral nutrient absorption, ultimately improving root growth. Furthermore, GABA has demonstrated tremendous potential in enhancing pomegranate growth even under non-stressed conditions. This study contributes to a better understanding of GABA’s role in the response of pomegranate plants to abiotic stresses, such as drought and salinity stress, and provides insights for successfully cultivating pomegranates under challenging environmental conditions at the farm level.

## Data Availability

The authors confirm that the datasets analyzed during the current study are available from the corresponding author upon reasonable request.
